# The systemic-level repercussions of cancer-associated inflammation mediators produced in the tumor microenvironment

**DOI:** 10.3389/fendo.2022.929572

**Published:** 2022-08-22

**Authors:** Dolores Aguilar-Cazares, Rodolfo Chavez-Dominguez, Mario Marroquin-Muciño, Mario Perez-Medina, Jesus J. Benito-Lopez, Angel Camarena, Uriel Rumbo-Nava, Jose S. Lopez-Gonzalez

**Affiliations:** ^1^ Laboratorio de Investigacion en Cancer Pulmonar, Departamento de Enfermedades Cronico-Degenerativas, Instituto Nacional de Enfermedades Respiratorias “Ismael Cosio Villegas”, Mexico City, Mexico; ^2^ Posgrado en Ciencias Biologicas, Universidad Nacional Autonoma de Mexico, Mexico City, Mexico; ^3^ Laboratorio de Quimioterapia Experimental, Departamento de Bioquimica, Escuela Nacional de Ciencias Biologicas, Instituto Politecnico Nacional, Mexico City, Mexico; ^4^ Laboratorio de Human Leukocyte Antigen (HLA), Instituto Nacional de Enfermedades Respiratorias “Ismael Cosio Villegas”, Mexico City, Mexico; ^5^ Clinica de Neumo-Oncologia, Instituto Nacional de Enfermedades Respiratorias “Ismael Cosio Villegas”, Mexico City, Mexico

**Keywords:** cancer, tumor microenvironment, inflammatory mediators, cytokines, systemic inflammation, paraneoplastic syndromes, systemic immune-inflammatory markers

## Abstract

The tumor microenvironment is a dynamic, complex, and redundant network of interactions between tumor, immune, and stromal cells. In this intricate environment, cells communicate through membrane–membrane, ligand–receptor, exosome, soluble factors, and transporter interactions that govern cell fate. These interactions activate the diverse and superfluous signaling pathways involved in tumor promotion and progression and induce subtle changes in the functional activity of infiltrating immune cells.

The immune response participates as a selective pressure in tumor development. In the early stages of tumor development, the immune response exerts anti-tumor activity, whereas during the advanced stages, the tumor establishes mechanisms to evade the immune response, eliciting a chronic inflammation process that shows a pro-tumor effect.

The deregulated inflammatory state, in addition to acting locally, also triggers systemic inflammation that has repercussions in various organs and tissues that are distant from the tumor site, causing the emergence of various symptoms designated as paraneoplastic syndromes, which compromise the response to treatment, quality of life, and survival of cancer patients. Considering the tumor–host relationship as an integral and dynamic biological system, the chronic inflammation generated by the tumor is a communication mechanism among tissues and organs that is primarily orchestrated through different signals, such as cytokines, chemokines, growth factors, and exosomes, to provide the tumor with energetic components that allow it to continue proliferating. In this review, we aim to provide a succinct overview of the involvement of cancer-related inflammation at the local and systemic level throughout tumor development and the emergence of some paraneoplastic syndromes and their main clinical manifestations. In addition, the involvement of these signals throughout tumor development will be discussed based on the physiological/biological activities of innate and adaptive immune cells. These cellular interactions require a metabolic reprogramming program for the full activation of the various cells; thus, these requirements and the by-products released into the microenvironment will be considered. In addition, the systemic impact of cancer-related proinflammatory cytokines on the liver—as a critical organ that produces the leading inflammatory markers described to date—will be summarized. Finally, the contribution of cancer-related inflammation to the development of two paraneoplastic syndromes, myelopoiesis and cachexia, will be discussed.

## Introduction

In 2020, GLOBOCAN estimated the global cancer statistics as 19.3 million new cases and 10 million deaths yearly (1). The increasing incidence and mortality rates reflect the growth and aging of the population and the increase in risk factors associated with socioeconomic development. Great efforts have been made to detect cancer early; however, most cases are detected at advanced stages.

Inflammation is a well-conserved process in which a distinct subset of cells from the innate and adaptive immune response is recruited to eliminate harmful agents in the host. This process is essential for the host’s defense against pathogens and is accompanied by tissue repair and wound healing to regulate tissue homeostasis. However, when dysregulated, inflammation contributes to the emergence and development of cancer. Tumor-associated inflammation is a well-recognized tumor-enabling characteristic that promotes or sustains the acquisition of some characteristics termed the hallmarks of cancer (2, 3). During tumor development, tumor-associated inflammation shapes the anti-tumor immune response towards a more permissive and pro-tumoral state (3). In this regard, the relationship between the tumor and the immune response is well known; according to immunoediting theory, at the early stages of tumor development, the immune system exerts anti-tumor activity through immunosurveillance (4). In this setting, as the tumor evolves, so does its microenvironment and the immune response, favoring the establishment of a pro-tumoral immune response. Several reports have indicated that the shift from anti-tumor immunity towards a pro-tumoral response is supported by a myriad of factors released from the tumor, immune, and stromal cells into the tumor microenvironment, which act to establish a persistent tumor-associated inflammatory state (5).

Nonetheless, the tumor-associated inflammatory state not only has repercussions in its immediate local microenvironment, but the release of various components into the bloodstream that promote or sustain inflammatory activity at the systemic level primes a cancer-induced systemic inflammatory response (6). At the plasma level, high concentrations of these proinflammatory factors can affect different organs or systems, such as the endocrine, nervous, dermatological, and hematological systems, among others, resulting in the alteration of the expression of some molecules or set of circulating cells, which are currently used as markers of systemic inflammation associated with cancer (5). In addition to the known cytokines, chemokines, and growth factors, it is now recognized that exosomes are one of the main factors capable of reaching different organs or systems, leading to the development of additional comorbidities called paraneoplastic syndromes (6). Among these paraneoplastic syndromes, neuropathy, hypercalcemia, dermatomyositis, cachexia, and dysregulated hematopoiesis cause detrimental effects on the patient’s quality of life and are sometimes manifested before cancer detection (7). In some instances, the clinical manifestation of paraneoplastic syndromes contributes to the promotion of tumor growth-promoting capabilities, leading to decreased overall survival (8, 9).

The study of cancer initially focused on the tumor’s genetic alterations and biological activity. Recently, the role of the bidirectional interactions between the tumor and its microenvironment as an integral and evolving biological system has been considered. Although human tumors are composed of heterogeneous cell populations, employing tumor cell lines and animal models has allowed us to deepen our understanding of the participation of the microenvironment throughout tumor development.

This review highlights the intricate signaling mediated by the different components released in the tumor microenvironment and their contribution at the systemic level. First, we will describe the interaction between the tumor and the immune cells and its evolution during tumor development. The local production of immune-stimulating factors by the stroma and immune inhibitory mediators induced or produced by the tumor will also be considered. At the systemic level, the effect of the main proinflammatory cytokines reaching their target organs and their impact on the production of inflammation markers will be addressed. Finally, the clinical manifestations associated with the development of inflammatory cytokines-induced paraneoplastic syndromes will be examined.

## Tumor microenvironment

According to the multistep carcinogenesis model, a tumor is shaped by a group of heterogeneous cells harboring genomic and epigenomic alterations. Transformed cells carrying driver mutations and epigenetic alterations activate aberrant signaling pathways that hinder the apoptotic process and promote uncontrolled cell proliferation. The growth of these transformed cells leads to changes in tissue architecture, which induces stress in the cells of the surrounding stroma, causing an increase in the production of soluble inflammatory mediators and growth factors and exosome release. These factors maintain a chronic inflammatory microenvironment that enables tumorigenesis (10). As the tumor grows, heterogeneous cell populations are generated due to the high and stochastic proliferation rate. Some of these new populations in the tumor mass acquire immune evasion mechanisms or produce soluble factors that modify immune cell phenotypes to support pro-tumor activity (11).

It has been recognized that the tumor microenvironment (TME) participates in cancer development and promotes the acquisition of some hallmarks of cancer (2). The composition of TME is heterogeneous; it is mainly composed of—but not limited to—cells such as endothelial cells, cancer-associated fibroblasts, pericytes, cancer stem cells, and immune-inflammatory cells, in addition to diverse extracellular matrix components (2).

In this context, the TME is a complex, redundant, and dynamic network that is constantly evolving throughout tumor development and progression. In this network, tumor, immune, and non-immune cells establish membrane–membrane and ligand–receptor interactions as well as communicate through the paracrine, juxtacrine, and internal secretion of various substances, such as proteins, different types of RNA, lipids, and biological mediators, which are delivered through the production of exosomes (12–14). Exosomes are vesicles between 40 and 160 nm in diameter. Exosomes arise from an early endosome in a process mediated by the endosomal sorting complex required for transport (ESCRT) (15). These mature endosomes are also known as multivesicular bodies (MVBs). MVBs can fuse with lysosomes for the degradation of their contents or can fuse with the plasma membrane, releasing their vesicles into the extracellular space (16). Exosomes can contain proteins, RNA, DNA, lipids, and carbohydrates. Initially considered as waste products of cells, exosomes are now known to play an essential role in cell communication (17). Most reports indicate that exosomes play paramount roles in tumor cell invasion, metastasis, and angiogenesis. In addition, exosomes are involved in modulating the TME, altering cellular metabolism, and promoting or inhibiting the immune response (18).

All of these interactions and molecule transfers activate diverse signaling pathways that affect gene expression, support the metabolic demands of different cell types, and induce the synthesis of various proteins that act as critical biomolecules to induce the participation of the immune response against genotoxic insults, incipient tumor formation, and tumor development (19, 20). During the early stage of tumor development, a nascent transformed cell develops in close interaction with the resident immune cells, among which the incipient transformed cell proliferates to form a small group of cells that lead to the distortion of the local tissue morphology. In this regard, and as part of the innate immune response, natural killer (NK) cells and resident macrophages eliminate susceptible tumor cells by releasing cytotoxic molecules that insert themselves into the tumor cell membrane, altering its permeability and causing cell death (21). Throughout this process, the dying cells expose molecules on their membrane or release intracellular molecules that acquire a new function, acting as alarmins or damage-associated molecular patterns (DAMPs) that promote the recruitment of other populations of immune cells, such as those involved in the adaptive immune response.

At this point, some reports have indicated that the exosomes released by tumor cells express class I and II MHC molecules and can prime and activate the immune response. As tumor cells develop and persistent growth occurs, the activation of the immune response continues and chronic inflammation is promoted, which initially stimulates an anti-tumor immune response (see below). However, it is known that chronic inflammation allows for the acquisition of new mutations and increased genome instability. Chronic inflammation causes the cellular composition of the tumor to become heterogeneous, resulting in a progressive change in the activities of the immune and stromal cells to promote a microenvironment that favors progression, invasion, and metastasis (22–24).

## Participation of the immune response in cancer

The relationship between chronic inflammation and cancer development is well known and is considered a hallmark of cancer (2). Virchow’s observations led him to propose that chronic inflammation provoked by the presence of an immune infiltrate was associated with the development of cancer (25). Afterward, Dvorak reported similar features between inflammation and cancer, such as proliferation, cell survival, angiogenesis, and migration (26).

The immune system is composed of an intricate network of cells, including NK cells, which are part of the innate lymphoid cells (ILCs) (27) and NKT cells, along with macrophages and dendritic cells (DCs), which are cells of the phagocytic mononuclear system that are involved in antigen presentation. As part of the adaptive immune response includes T lymphocytes, such as CD4+ T and CD8+ T cells, and B lymphocytes (28). The detailed study of tumor-infiltrating immune cells in biopsied material obtained from cancer patients has indicated that immune cells interact with tumor cells through the production of diverse factors, such as cytokines, chemokines, the by-products of cell metabolism, growth factors, and the components of exosomes, which participate during the tumor development stages (29). It has been suggested that immune cells and the soluble factors they secrete induce a particular microenvironment that, in the early stages of tumor development, supports anti-tumor activities; nevertheless, the microenvironment evolves, and in the advanced stages of the tumor, the immune cells are modulated to promote tumor growth (29).

According to emerging knowledge on the biological role and physiological importance of the different cells that compose the immune system, it has been proposed that NK cells patrol the human body to recognize normal self-cells, a process carried out by two types of receptors. Thus, NK cell activation is tightly regulated by an intricate balance between activation and inhibition signals (30, 31). In a normal cell, the peptides derived from self-proteins are loaded onto class I MHC molecules and are recognized by NK cells through the killer cell immunoglobulin-like receptor (KIR). In contrast, the recognition of self-cell ligands, such as the stress-induced proteins MICA, MICB, and ULBP-1, is mediated by the natural cytotoxic receptor (NCR) (32, 33). In tumor cells, tumoral peptides are associated with class I molecules, impeding recognition by KIR receptors and triggering effector activity. For full activation, NK cells depend on glycolysis and oxidative phosphorylation (OXPHOS), which are modulated by mTORC1 (34, 35). NK cells fight tumors by releasing cytolytic molecules, such as perforin, granzymes, and granulysin, causing the death of sensitive tumor cells. Some authors have also shown that NK cells can release exosomes containing these cytolytic molecules that reduce or eliminate malignant cells in both tumor-bearing animal models and human tumor cell lines of distinct origins (36–38).

In addition, activated NK cells release several soluble mediators, such as tumor necrosis factor-alpha (TNF-α); interferon-gamma (IFN-γ); interleukin (IL)-10; chemokines, including CCL3, CCL4, CCL5, XCL1, etc.; and growth factors, such as granulocyte macrophage colony-stimulating factor (GM-CSF), etc. (39). IFN-γ is known to be essential for immune cell activation; in NK cells, it increases cell activity and cytolytic potential (40, 41). From this point of view, NK cell overactivity increases the proportion of dying tumor cells and releases more DAMPs are released, which act as “find me” signals, and tumor antigens. These tumor-released compounds promote the arrival of inflammatory and immune cells, initiating an acute inflammatory process (42, 43). In this setting, tissue-resident macrophages, dendritic cells (DCs), and recruited monocyte-derived DCs comprise the mononuclear phagocytic system (44, 45), playing a critical role in homeostasis, tissue repair, the immune response, and cancer (46). In local tissues, resident and immature DCs (iDCs) exhibit elevated phagocytic activity mediated by the expression of a variety of pattern recognition receptors (PRRs) (47), which recognize the DAMPs and tumor antigens released from dead and dying tumor cells. Then, the iDCs trigger a rigorous metabolic process to meet the cell’s energy demands, including increased aerobic glycolysis, decreased OXPHOS with a concomitant increase in nitric oxide (NO) production, and increased fatty acid (FA) metabolism (48, 49). ROS production regulates the acidification of the lysosomal compartment for the degradation of phagocytosed antigens to peptides, while FA metabolism supplies the components for cell membranes. During these events, the endoplasmic reticulum and Golgi apparatus are expanded for protein synthesis, which assists in the upregulation of class II MHC molecules and antigen cross-presentation by class I MHC molecules; the expression of the costimulatory molecules CD80, CD86, and CD40; the expression of receptors for chemokines; and cytokine secretion, including interleukin (IL)-1, TNF-α, IL-6, IL-8, IL-12, IL-15, IL-18, etc. All of these activities induce the progressive maturation of DCs to become professional antigen-presenting cells (APCs). Then, the APCs travel to the lymph node through the lymphatic vessels, a process in which glucose metabolism plays a critical role (50, 51).

In the lymph node, DCs (mDCs) act as potent APCs that stimulate the proliferation and maturation of naïve antigen-specific CD4+ T cell clones and, by antigen cross-presentation, the activation of naïve antigen-specific CD8+ T cells. In addition to direct cell–cell interactions, some studies have indicated that exosomes released by APCs can also induce T-cell activation. It has been demonstrated that they express peptides associated with class I and II MHC and costimulatory molecules. In addition, they can also activate T and NK cells through the NKG2D–NKG2D ligand interaction (52–58).

Soon after the initial T-lymphocyte priming, T cells upregulate aerobic glycolysis, increasing glucose transporters and enzymes to meet their energetic demands; glutaminolysis and increased amino acid uptake favor OXPHOS for ROS and NO synthesis. In addition to mitochondrial biogenesis, lipogenesis by the endoplasmic reticulum and Golgi apparatus are required. Following this PI3K-AKT-mTORC1-dependent metabolic reprogramming, effector CD4+ T cells secrete several cytokines, such as IL-2, IFN-γ, etc., that induce the activation of specific transcriptional programs for the stimulation of antigen-specific CD8+ T cells and the overactivation of NK cells (59–61). The CD8+ T cells then release various cytokines, such as IFN-γ, IL-2, and TNF-α, and synthesize cytolytic molecules to become effector cytotoxic T lymphocytes (CTLs) (60, 62). After T-cell expansion, effector CD4+ T cells and CTLs migrate through the bloodstream and infiltrate the tumor, becoming critical cells for tumor destruction (63). A recent report from Rezaei R et al. using a CT-26-induced BALB/c mouse model of colorectal cancer indicated that when incorporated into tumor-derived exosomes, miR-124-3p, which acts as a post-transcriptional regulator of gene expression, stimulates a potent antitumor immune response, diminishing T regulatory (Treg) cells, reducing tumor mass, and increasing the overall survival rate (64). This miRNA is downregulated in colon cancer compared to non-malignant tissue, and *in vitro* studies have indicated that in Treg cells, PD-L1 expression is inhibited by cytokines such as TGF- β and IL-10 (65). All of this information suggests the possible involvement of exosomes released by tumor cells in the induction of a potent anti-tumor immune response. The cytokines released by T cells create a positive feedback loop that perpetuates the inflammatory process, as the array of pro-inflammatory cytokines leads to the overstimulation of innate immune cells. In addition, the overstimulated NK cells upregulate the activity of tissue-resident macrophages and the recruited neutrophils at the tumor site. These phagocytic cells carry out the respiratory burst to further produce pro-inflammatory cytokines and release ROS and NOS, promoting the M1 and N1 cell phenotypes, respectively. The induced anti-tumor activity leads to the additional destruction of tumor cells (66). Exosomes released by these metabolically activated cells mimic the tumoricidal activity of M1 and N1 cells (67–69).

A chronic inflammatory process is induced when this cellular circuit is maintained to eliminate tumor cells which mutations generate immunogenic changes in the synthetized tumor proteins. Reports have indicated that the chronic inflammatory process causes the release of transferrin-bound iron, which accumulates in the extracellular space. It is known that tumor cells take up this element, which promotes the production of DNA-damaging ROS. Increased DNA damage may lead to cell death in some vulnerable tumor cells in a process known as cell death mediated by ferroptosis (70). Conversely, these and other mutation-causing factors could promote genomic instability and epigenetic changes in other cells within the tumor cell population, which could lead to the maintenance of cell viability and enhance tumor proliferation, induce resistance to apoptosis, and, concomitantly, increase tumor heterogeneity (71).

Thus, the tumor comprises new tumor cell clones, increasing phenotypic heterogeneity, and the tumor mass itself. Oncogenic changes promote the activation of various signaling pathways in the heterogeneous tumor population, increasing the release of exosomes with different molecules or soluble factors that reinforce the inflammatory phase of chronic inflammation. For example, driver mutations in genes, such as *MYC, K-RAS*, or *RET* activate signaling pathways that promote the synthesis and release of proinflammatory cytokines, such as IL-8, IL-1, and CXC chemokines (72–74).

Depending on the tumor type, stage of tumor progression, genetic background, clinicopathological characteristics of the patient, etc., the distribution and density of non-malignant cells infiltrating the tumor vary greatly. Tumor and non-malignant cells produce several cytokines, growth and differentiation factors, chemokines, lipids, and nucleic acids that are released or loaded into exosomes, generating a wide array of molecules that promote cancer. Various research groups have published excellent reviews describing the signaling pathways involving cytokines, growth factors, and exosome components that may play a role in cancer (14, 18, 54, 75–77).

In the more advanced stages of cancer development, the surrounding tumor, stromal, and immune cells show a high rate of proliferation that requires high metabolic activity, resulting in the release of various by-products. In addition to the factors released by the various cells that make up the tumor, these metabolic by-products create a complex and changing microenvironment, which gradually promotes cancer cell survival and tumor mass growth. High metabolic activity is indispensable due to the chronic inflammatory process induced by the tumor. The increased energy and biosynthetic requirements mediated by increased glucose uptake and aerobic glycolysis favor the tumor’s proliferation, differentiation, and growth and affect the stromal cells. During this metabolic reprogramming, tumor cells produce—or induce the immune and stromal cells to produce—several cytokines that stimulate tumor growth while inhibiting or blocking the effector activity of immune cells. Some of the cytokines produced include IL-10, IL-6, IL-4, High Mobility Group-Box 1 (HMGB1) protein, etc., while several growth factors are generated as well, such as Epithelial Growth Factor (EGF), vascular endothelial growth factor (VEGF)-A, transforming growth factor-β (TGF-β), platelet-derived growth factor subunit A (PDGF-A), angiopoietin-like 4 (ANGPTL4), etc.; information about these factors is summarized in **Table 1**. In addition, some chemokines and their receptors play an important role in the TME and are expressed by tumor, immune, and stromal cells. Due to their anti- and pro-tumor effects, α-chemokines, i.e., CXC chemokines that contain a CXC motif at their N-terminus, have attracted attention. Some reports have indicated that CXCR3 and its corresponding ligands, CXCL14 and CXCL16, recruit primary immune cells with immune regulatory functions and pro-tumor activities, such as tumor-associated macrophages (TAMs) and neutrophils (TANs), myeloid-derived suppressor cells (MDSCs), and Treg cells (120–122). Chemerin was initially described as a chemotactic factor for NK cells, macrophages, and myeloid and plasmacytoid DCs (123–125), favoring tumor infiltration by leukocytes and the regulation of cell metabolism. Current reports indicate that different cell types produce chemerin, including fibroblasts, epithelial cells residing in the tumor niche, and cells from distant organs, such as hepatocytes and adipocytes (126–128). Although most tumors downregulate the expression of chemerin, the potential pro- and anti-tumor activities of this molecule have been reported in the TME and have been suggested as a prognosis marker (129). More information on this topic is beyond the scope of this review; however, further information can be obtained from previous studies (130, 131).

**Table 1:** Cytokines and Growth Factors associated with cancer-related inflammation.

Cytokine	Primary Target Cell	Biological activity in cancer	Ref.
IFN-γ	Macrophages, NK, and T-cells	Up-regulates expression of MHC-I and-II molecules and antigen presentation. Inhibits proliferation of tumor cells and induces necroptotic cell death.	(78–80)
IL-1	NK, T-, M1 macrophages, and tumor cells	Promotes systemic and local inflammation. Facilitates angiogenesis through activation of endothelium and metastasis. Participates in mobilization of HSPCs in bone marrow to yield MDSCs.	(81, 82)
IL-2	T-CD4/CD8 and NK cells	Drives the activation of tumor-infiltrating CD8+ T cells.	(83)
IL-4	Th2 cells, basophils, eosinophils, and macrophages	Decreases the activity of TAM and CD8+ T cells. Induce the expression of Th2 cytokines modulating the antitumor immune response. Induce a regulatory phenotype on NK cells by modulating DCs. Stimulates the growth of tumor cells and cell death resistance.	(84–88)
IL-6	Monocytes, macrophages, endothelial cells, B- and T-cells, and tumor cells	Regulation of acute phase response, activation of T helper cells. Promotes the growth of tumor cells and favors their survival. Implicated in angiogenesis.	(89–91)
IL-8	Neutrophils, endothelial cells, and pericytes	Attraction of MDSCs into the tumor. Activation of angiogenesis. Regulation of stem cell properties.	(92, 93)
IL-10	T-, B-, dendritic cells (DCs), Th2 lymphocytes, Tregs, and macrophages	Inhibits the expression of MHC class I and II molecules and antigen presentation in APCs and tumor cells. Contribute to immunosuppression by hindering the effector activity NK, Th1, and CD8+ T cells. Negatively correlates with tumor-infiltrating CD8+ IFN-γ+.	(94–97)
IL-12	NK, APCs, and T-cells	Promotes proliferation and cytotoxic effect of NK cells. Enhance the anti-tumor activity of M1 and Th1 cells.	(98)
IL-17	Mucosal tissues, fibroblast, epithelial, endothelial, Th17, NK cells, and monocytes	Contributes in tumor growth, metastasis and cancer-related inflammation.	(99, 100)
IL-18	Th1, NK, DCs, macrophages, keratinocytes, and B cells	Pro-inflammatory cytokine. Cooperates with IL-12 inducing IFN-γ production from T helper and NK cells, leading to NK cell activation; up-regulates antigen presentation and exhibits antiviral and antitumoral functions. Suppress tumor growth by downregulating VEGF production within tumor.	(101, 102)
TNF-α	Neutrophils, macrophages, monocytes, and endothelial cells	Increase tumor cell growth, angiogenesis, and metastasis. Participates in promoting cancer-associated inflammation.	(103)
TGF-β	MDSCs, Tregs, and tumor cells	Increase the expression of PD-1 on intra-tumoral CD8+ T cells resulting in their dysfunction and exhaustion. Inversely correlates with the frequency of CD8+ T cells in the tumor niche. Suppress the cytotoxic activity of NK cells. Promotes the activation of the EMT program.	(104–107)
GM-CSF	Lymphocytes, macrophages, fibroblast, endothelial cells, and tumor cells	Promotes DCs differentiation, in response to cytokine or inflammatory stimuli, activates the effector functions of myeloid cells at the resolution of inflammation to promote wound healing and tissue repair.	(108)
G-CSF	Fibroblast, stromal cells, monocytes, macrophages, and endothelial cells	Stimulates extramedullary hematopoiesis in the liver. Causes the differentiation of HPSCs into myeloid precursors in bone marrow. Recruits DCs and activates Tregs and secretion of Th2 cytokines.	(109, 110)
PDGF	Platelets, macrophages, osteoblasts, fibroblasts, and tumor cells	Chemoattractant of fibroblasts. Stimulates angiogenesis and activation of EMT.	(111–113)
VEGF	Smooth muscle cells, keratynocytes, platelets, endothelial cells, neutrophils, macrophages, and tumor cells	In endothelial cells induces a mitogenic effect and resistance to cell death. Promotes apoptosis of CTLs through Fas-FasL in tumor vasculature. Hampers the maturation of DCs.	(114–116)
EGF	Epithelial cells, fibroblast, platelets, endothelial cells, glands, and tumor cells	Over-expression correlates with TGF- β, tumor growth, metastasis, and resistance to anti-tumor agents	(117–119)

In the TME, the high proliferation rate of tumor cells induces hypoxia, leading to hypoxia-induced acidosis caused by the release of lactate (132). During this metabolic reprogramming in liver cancer, the lncRNA HULC modulates the activity of crucial glycolytic enzymes through phosphorylation (133). In addition, LINC00261 promotes aerobic glycolysis in pancreatic cancer by activating the miR-222-3p/HIPK2/ERK axis (134). The participation of exosomes during hypoxia is an emerging research area. In one study, the long-intergenic non-coding RNA regulator of reprogramming (LINC-RoR) contained in the exosomes released by hepatocellular cancer cells under hypoxic conditions was shown to lead to increased HIF-α expression and the poorer survival of patients (135). In addition, in colorectal cancer, LINC00152 has been shown to be released in exosomes under hypoxic conditions, participating in the pathogenesis and progression of this cancer (136, 137). The production and secretion of lactate by tumor and infiltrating immune cells induce a gradual reconversion of immune cells from anti-tumor to pro-tumor activity to promote tumor immune evasion and support the migration and invasion of tumor cells (138, 139).

Tumor cells increase their iron uptake to supply their bioenergetic demand. Iron metabolism impacts DNA synthesis, cell cycle progression, and morphogenesis in physiological processes during normal cell life. This metabolic program also participates in invasion, metastasis, and EMT (140). However, as mentioned previously, increased cytoplasmic iron levels could induce a type of cell death known as ferroptosis (141). Cells undergoing ferroptosis release DAMPs associated with cell death, such as HMGB1, ATP, etc. (142). In the TME, extracellular ATP released by dead or dying cancer cells is hydrolyzed by several families of ectonucleotidases (143), mainly CD39 and CD73, which are expressed by immune and endothelial cells (144, 145). These enzymes are responsible for the conversion of extracellular ATP to adenosine. Adenosine accumulation in hypoxic cancer tissue is sensed by A2AR and A2BR receptors on immune cells that hamper the anti-tumor immune response. It is known that A2AR blocks the immune cells secretion of IFN- γ and IL-2. At the same time, A2BR prevents antigen presentation by mDCs and induces the polarization of M1 to M2 macrophages and the stimulation of MDSCs (146, 147).

Additionally, metabolic reprogramming activates signaling pathways that lead to aberrant gene expression due to epigenetic changes that alter tumor and stromal cells. These metabolic changes produce cells that also display the pro-tumor properties, such as cancer-associated fibroblasts (CAFs), induced by the TGF- β and EGF secreted by the tumor (148). In addition, they produce several chemokines; TGF- β ; IL6; some growth factors such as hepatocyte growth factor, insulin-like growth factor, etc.; and release exosomes. Furthermore, this environment favors the recruitment of MDSCs (149), TAMs (150), mesenchymal stem cells (MSCs) (151), and Tregs (152, 153). The role of exosomes in promoting the participation of these subsets of immune cells displaying pro-tumoral activity is discussed in the following reviews (154, 155).

**Figure 1:**
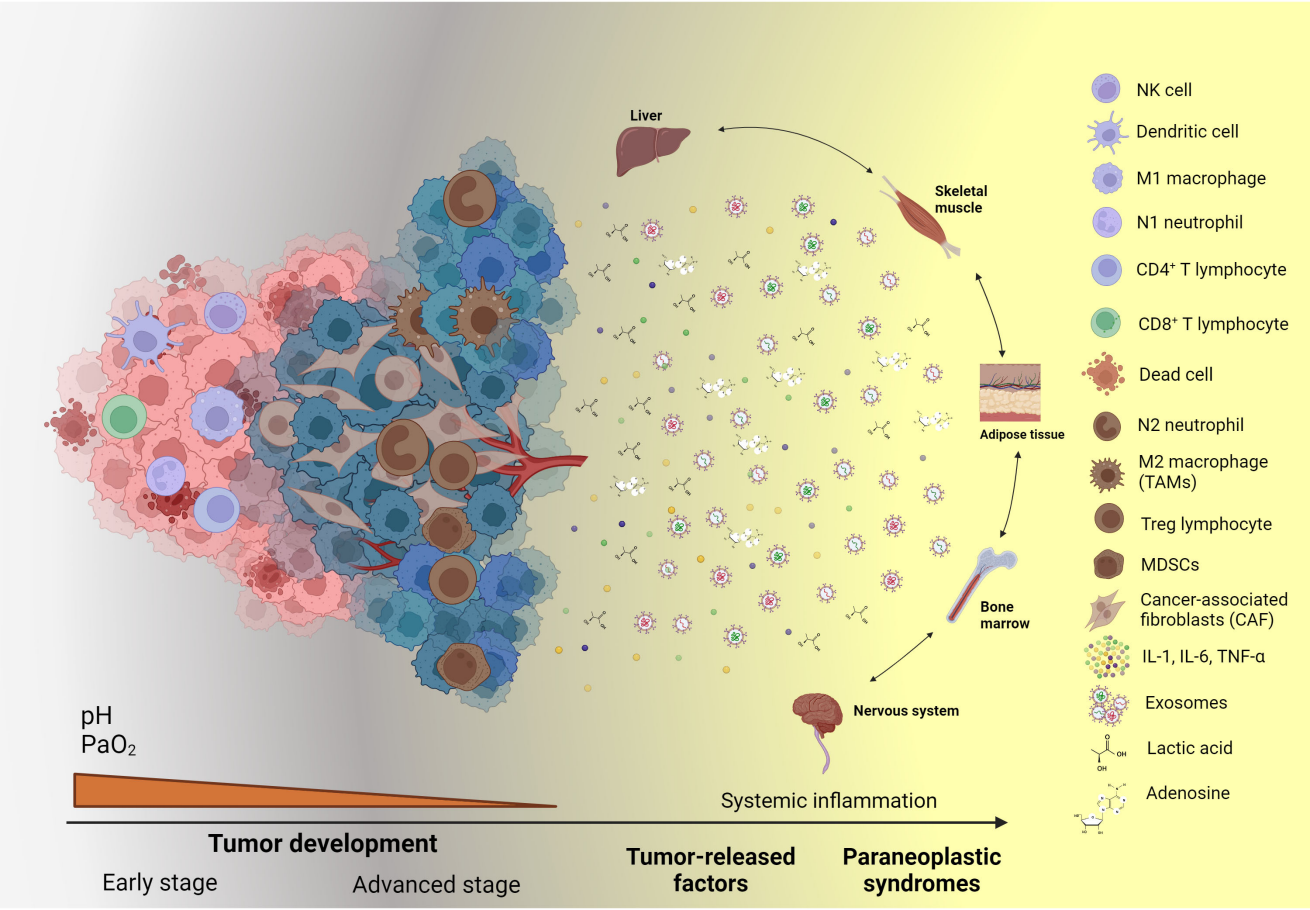
Systemic effects and paraneoplastic syndromes caused by cancer-associated inflammation. During the advanced stages of tumor development, tumor and stromal cells release an array of soluble factors, such as cytokines, chemokines, growth factors, metabolic by-products, exosomes, and ncRNAs, which sustain the local inflammatory state. Moreover, these soluble factors, when released into the bloodstream, reach distinct organs, systems, and tissues, causing alterations in their function and the production of diverse molecules and subsets of cells, which can be employed as biomarkers to assess cancer-related systemic inflammation. Created with BioRender.com.

In the TME, the accumulation of cytokines, chemokines, soluble factors, and a mixture of biomolecules in exosomes compromises the immune response during the advanced stages of cancer (156–158). Soluble factors with immunosuppressant activity, such as IL-10, TGF-b, IL-4, IL-6, etc., impair the function of NK cells (159, 160) and CTLs (161, 162) by inhibiting their effector activity and downregulating the expression of transcripts coding for cytolytic molecules. This cytokine environment induces the differentiation of CD4+ T to Treg cells (163–165) and maintains a stage of dedifferentiation in MDSCs by enhancing the expression of TGF-β and IL-10. In addition, tumor-resident MDSCs produce cyclo-oxygenase 2 (COX-2), arginase 1 (ARG1), inducible NO synthase (iNOS), IL-10, and indoleamine 2,3-dioxygenase (IDO), which accentuate the suppressor milieu (166). In hypoxia, pyruvate is reduced to lactate, which decreases the local pH. Moreover, IL-4 or IL-13 stimulates the polarization of the macrophages from the M1 to M2 phenotype to support tumor progression and angiogenesis (167) (**Figure 1**).

## The systemic effect of tumor microenvironment-derived cytokines

Studies performed with cancer patients and animal models have studied several local and systemic cytokines. In cancer patients, diverse cytokines and soluble factors showing pro- and anti-inflammatory activities may be detected as free circulating or exosome membrane-bound molecules (168). Reports have indicated that the cytokines IL-1, TNF-α, IL-6, G-CSF, and GM-CSF mainly act at the systemic level, affecting the function of some organs (169). Our group quantified eight cytokines at the systemic level in smoking patients with lung adenocarcinoma and found a significant increase in IL-2, IL-4, IL-6, and IL-10 compared to their levels in healthy smoking subjects. Among these cytokines, the concentration of IL-6 was the highest in the peripheral blood of cancer patients, showing an increase of approximately sevenfold. To explain the increased systemic levels of IL-6, several reports from distinct groups, including ours, have pointed out that tumor cells from different types of cancers produce this cytokine (170, 171). Currently, the roles of these cytokines in cancer have gained considerable attention. In particular, those cytokines acting at the systemic level have been associated with the development of some clinical signs or symptoms of paraneoplastic syndromes.

### TNF-α

As discussed in the previous section, the tumor and its stroma release cytokines that act on various systems and organs. TNF-α is a pro-inflammatory cytokine with diverse functions that participates in homeostatic and distinct pathological conditions. The biological activity of TNF-α is exerted *via* binding with its cognate receptors: TNF-α receptor 1 (TNFR1) and 2 (TNFR2). TNFR1 is ubiquitously expressed in cells and is activated by the transmembrane or the soluble form of TNF-α. To the contrary, the expression of TNFR2 is limited to specific cells, such as immune and endothelial cells and cells of the central nervous system, and its activation mainly depends on the transmembrane form of the ligand (172). The role of these receptors in homeostasis has been described. The activation of TNFR1 mainly promotes inflammation, the induction of cell death *via* apoptosis or necroptosis, and tissue degeneration.

Conversely, the activation of TNFR2 is associated with cell survival and the wound healing process (172). Once TNF-α is produced and released into the bloodstream, this cytokine can reach the adipose tissue, where it modifies the adipocytes glucose and lipid metabolism. In this regard, TNF-α decreases glucose uptake by inhibiting the signaling pathways triggered by insulin and, as a result, downregulates the mRNA and protein expression of GLUT-4 (173, 174). Simultaneously, TNF-α activates lipolysis *via* the inhibition of the peroxisome proliferator-activated receptor (PPAR)- γ and CCAAT enhancer-binding protein, causing an increase in the expression of neutral lipases (175). These observations might explain the increased levels of serum lipids detected in cancer; however, more studies are required to demonstrate the role of TNF-α during the dyslipidemia observed in cancer (176).

Studies in cancer patients have demonstrated that cancer-associated systemic inflammation is associated with a sense of pain or hyperalgesia from an unknown source. In peripheral nerves, TNF-α is linked with this phenomenon. In this setting, the interaction of TNF-α with TNFR1 causes the activation of the p38/MAPK signaling pathway, which culminates in the activation of Na+ and K+ ionic channels in peripheral nerves, leading to pain generation (177, 178).

In addition, the findings from a recent study indicated that in hepatocellular carcinoma, TNF-α released in exosomes promoted osteoclast differentiation. During this phenomenon, the TNF-α stored in the exosomes produced by hepatocellular Huh-7 cells caused the expression of osteoclast-associated differentiation markers when added to murine macrophage/monocyte cell lines through the activation of the NF-κB/cathepsin K/triiodothyronine receptor auxiliary protein axis. These findings explain the tendency of hepatocellular cancer cells to generate bone metastases (179).

### Interleukin-1

IL-1 is a pleiotropic cytokine that is involved in various inflammatory processes. IL-1 belongs to the Ig-like receptor superfamily, which is characterized by the presence of the Toll/interleukin-1 receptor (TIR) domain. The TIR domain is essential for the biological activity of IL-1 (180, 181). The ligands of the IL-1 family are IL-1, IL-18, IL-33, and IL-36. These agonists bind to three receptors: IL-1α and IL-1β perform cell activation when bound to IL-1R1; IL-18 is a ligand of IL-18Ra; IL-33 binds to ST2 (IL-1R4); and IL-36α, β, and γ are agonists of IL-1Rp2 (IL-1R6). The primary function of this family of ligands and receptors is to participate in inflammatory processes (182, 183).

IL-1α and IL-1β are encoded by different genes and have minimal homology. IL-1α is synthesized as pro-IL-1α, which can be proteolytically cleaved by calpain, granzyme B, elastase, and chymase. When the mature form of IL-1α is released, it acts as an “alarmin,” activating the innate immune response against noxious stimuli (184). IL-1β is a critical player in the inflammasome. The activation of the inflammasome in innate immune response cells, such as monocytes and neutrophils, occurs when pathogen-associated molecular patterns (PAMPs) and DAMPs bind to Toll-like (TLRs) and NOD-like (NLRs) receptors. The assembly of the inflammasome causes the cleavage of pro-caspase 1, and caspase 1 is released, which then cleaves pro-IL-1β and pro-IL-18. IL-1α and IL-1β establish chronic inflammation in the process of carcinogenesis (185). In IL-1/IL-1R1 knockout murine models, it was observed that IL-1 was a critical factor in the inflammatory process associated with 3-methylcholanthrene carcinogenesis (186). Both IL-1α and IL-1β participate in the systemic inflammation associated with cancer. Tumor and stromal cells in the tumor microenvironment produce and release IL-1.

Recent reports have indicated that cancer-derived exosomes from prostate and lung cancer as well as glioblastoma cell lines stimulate the production and release of IL-1β in immune and non-immune cells (187–189). In this setting, cancer-derived exosomes may trigger distinct intracellular signaling pathways in receptor cells, culminating in NF-κB activation and the subsequent expression of the *IL-1β* gene or the activation of inflammasomes through NLRP3 for the cleavage of pro-IL-1β into its active form (190, 191). In support of this, Linton et al. reported that exosomes derived from pancreatic ductal adenocarcinoma (PDAC) cell lines caused the polarization of M0 into immunosuppressive pro-tumoral M2 macrophages, which increased the production and release of IL-1β (327). In this regard, PDAC-derived exosomes were shown to contain increased levels of arachidonic acid, leading to its subsequent metabolism into free fatty acids, which activate inflammasomes through NLRP3 (193). Several reports have indicated that IL-1 in circulation affects several organs and may contribute to establishing paraneoplastic syndromes, such as cachexia (see below) (194–197).

### IL-6

Low blood levels of IL-6 ranging between 1 and 5 pg/mL have been reported under normal conditions (198). In addition, soluble forms of the receptors IL-6R and gp130 have been detected. After local production by tumor cells in the inflammatory niche, IL-6 is released into the bloodstream and eventually reaches the liver, where it has several biological effects on hepatocytes. In one study, an increase in the production of acute phase proteins (APPs), such as C-reactive protein (CRP), serum amyloid A (SAA), fibrinogen, haptoglobin, and α1-antichymotrypsin, and an opposing decrease in albumin, fibronectin, and transferrin were detected. In addition, IL-6 regulates the transporters of iron and zinc associated with anemia that are detected in chronic inflammation (199). IL-6 also promotes megakaryocyte maturation in the bone marrow, increasing the serum platelet count (200).

The varied effects induced by IL-6 are detected mainly in chronic inflammatory diseases. In addition, this cytokine exerts various biological actions on the distinct types of immune cells, maintaining a deregulated and persistent positive feedback loop. IL-6 has evident pleiotropic effects in hematopoiesis, inflammation, the immune response, and cancer.

## Parameters to assess systemic immune-inflammatory markers

The liver is a primordial organ that eliminates waste and toxic compounds, mainly found in dietary products, or harmful particles from pathogens. In addition, it provides nutrients and produces mediators that alert immune cells to induce an inflammatory response that eliminates harmful agents and induces the restoration of tissue homeostasis. The products of pathogens (PAMPs) or those derived from the host’s damaged cells (DAMPs) induce the release of acute phase proteins from the liver (201, 202). Kupffer cells and macrophages produce proinflammatory cytokines, such as IL-6, IL-1β, and TNF-α, to generate a series of products associated with the inflammatory response (203, 204). These cytokines activate resident cells, such as hepatocytes, endothelial cells, hepatic stellate cells, and diverse immune cells, from the hepatic arteries and portal vein in the liver. In addition, the liver releases diverse enzymes that inactivate harmful drugs and produces serum proteins, such as albumin and coagulation factors (205) (**Figure 1**).

Local inflammation appears to be reflected at the systemic level, and this is supported by several studies (6, 206, 207). Routine hematological parameters have been used in recent years as indicators of systemic inflammation. Among these simple parameters, the proportions of circulating inflammatory cells, including the white blood cell (WBC) count, lymphocyte count, neutrophil count, platelet count (PLTs), mean platelet volume (MPV), and levels of hemoglobin (Hb) and serum CRP, have been screened (208, 209). Analyte-based scores or ratios of some of these parameters have been reported to assess systemic inflammation, and their correlation with the prognosis of numerous pathologies, including cancer, has been described.

The Glasgow prognostic score/modified Glasgow prognostic score (GPS/mGPS) is an inflammatory indicator (210, 211). The GPS/mGPS reflects the systematic inflammatory response and nutritional status. Recent studies have shown that the GPS/mGPS is a novel inflammatory index that can predict outcomes in various cancers (212–214). However, the molecular mechanisms underlying the relationship between the GPS/mGPS and poor prognostic outcomes are still unclear. A plausible explanation is that an elevated GPS/mGPS may reflect an individual’s immune and nutritional status. The GPS/mGPS comprises albumin and CRP; both are acute-phase proteins synthesized in the liver. The CRP level is regulated by several pro-inflammatory cytokines, such as IL-1, TNF-α, TGF-β, IFN-γ, and IL-6 (215). Studies have shown that IL-6 correlates with OS and SOC in CRC, and its effect could explain the promotion of tumorigenesis and metastasis (216, 217).

Additionally, CRP is associated with the activity of infiltrated immune cells, including DCs, T cells, and NK cells (218, 219). Many studies have shown that CRP is an independent biomarker for predicting prognostic outcomes in various cancers (220, 221). The serum albumin level is used to evaluate liver function and nutritional status. Hypoalbuminemia is a common feature of the systemic inflammatory response, cancer recurrence, and metastasis. In addition, it has been shown to be positively correlated with the OS and CSS of patients with various cancers, including CRC and ovarian cancer (222, 223).

### Albumin

Albumin is a low-molecular-weight protein of 66 kDa consisting of a single polypeptide chain with 585 amino acid residues that is fully synthesized in the liver, which produces approximately 15 g daily. Albumin maintains a plasma concentration of 35–45 g/L and is the most abundant protein in plasma, contributing to the maintenance of oncotic pressure and the permeability of the microvasculature. In addition, it has been implicated in important metabolic functions as it transports several endogenous ligands, such as free FAs, bilirubin, and ion metals, as well as some exogenous ligands (224, 225). Albumin expression is mainly regulated at the transcriptional level. Decreased albumin synthesis leads to hypoalbuminemia, which contributes to the development of edema by the transudation of fluids into extravascular spaces. TNF-α is a key cytokine involved in the inhibition of albumin synthesis, although other cytokines, such as IL-1β and IL-6, may contribute as well.

The serum albumin level is a marker of nutritional status, and a level less than 35 g/L is considered to indicate hypoalbuminemia. Albumin acts as an anti-inflammatory molecule; its increase is associated with blocking the migration of neutrophils through the endothelium by decreasing the expression of VCAM-1 in a TNF-α-dependent manner (226). Regardless of the disease, decreased serum albumin has been proposed as a risk factor and predictor for morbidity and mortality.

### Platelets

Platelets, or thrombocytes, are nonnucleated and discoidal fragments derived from precursor megakaryocytes during megakaryopoiesis. Platelets maintain normal hemostasis and participate in several biological processes, such as the control of blood vessels and their interactions with endothelial cells. They also form a platelet plug with various extracellular matrix components, which inhibits vascular leakage. In addition, they are vital in acute and chronic inflammation due to their release of cytokines and chemokines that attract leukocytes and favor immune cells to reach the damaged tissue for wound healing. Furthermore, they are critical participants in the pathophysiology of several diseases, including cancer (227–229). Platelet characteristics have been found to be significantly associated with the clinical outcomes of several pathologies. Two of these features are the number of circulating platelets, designated as the platelet count, and the mean platelet volume (MPV).

The complete blood count includes the platelet count and the MPV. The platelet count quantifies the number of platelets in the blood; there are usually between 150,000 and 450,000 platelets in each microliter. Platelet parameters are used in the diagnosis of a patient’s general condition and have a prognostic value in some pathologies. A platelet count is related to pathologies associated with a chronic condition (229). MPV is a measure of platelet size that results from a higher production by megakaryocytes; hence, it indicates if there are more young platelets circulating in the bloodstream. The MPV ranges between 7.5 and 12.0 fl (229). The value is inversely related to the platelet count, hemostasis maintenance, and the preservation of a constant platelet mass. MPV is a marker of platelet activity, and it has been related to prothrombotic and proinflammatory diseases (230).

Under inflammatory conditions, the increase in IL-6 causes an increase in the ploidy of megakaryocytic nuclei and an increase in the cytoplasmic volume, leading to the generation of a large number of platelets. These platelets migrate to the site of inflammation, where they undergo activation and are depleted, triggering a decrease in the MPV of the patient’s blood during the development of the inflammatory process (229).

### Fibrinogen

Fibrinogen is produced in the liver during general acute-phase inflammatory provocation. Inflammatory cytokines induce fibrinogen synthesis by hepatocytes and Kupffer cells. Several reports have indicated that IL-6 is a key cytokine that promotes fibrinogen production due to the presence of several IL-6 response elements in the fibrinogen genes. In addition, IL-6 regulates fibrinogen transcripts *via* the MEK-ERK signaling pathway (231, 232).

In association with other molecules, fibrinogen participates in coagulation, fibrinolysis, and cellular and matrix interactions that support cell migration, inflammation, and wound healing. An increased risk of cardiovascular disease has been associated with elevated fibrinogen levels (233). Cancer patients present with malfunctions in the coagulation process, and this molecule has been associated with cancer development. Fibrinogen binds to growth factors, such as fibroblast growth factor-2 (FGF-2, bFGF) and (VEGF, to enhance tumor growth and increase the migration, invasion, and metastasis of tumor cells, as well as angiogenesis—processes which are considered to be hallmarks of cancer. The fibrinogen/albumin (F/A) ratio is considered a promising inflammation-based marker. A high FAR was shown to be associated with clinical–pathological features and survival in some cancers (234). However, multicentral clinical trials are needed to analyze the clinical impact of this relationship on medical oncology.

The evidence presented thus far provides an explanation for the usage of these hematological parameters and their diverse relationships to analyze the systemic inflammatory state. These ratios are associated with prognosis and survival in inflammatory diseases, including cancer (235). Owing to the extensive information indicating the prognostic value of these ratios, we only refer to those works in which these indices were assessed in the most frequent types of cancer. The reported ratios are indicated in **Table 2**.

**Table 2:** Available systemic inflammation indices predicting prognosis and outcome in cancer patients.

Index	Calculation	Ref.
Glasgow prognostic score (GPS)	CRP ≤ 10 mg/L and albumin ≥35 g/L Score 0 CRP ≤ 10 mg/L and albumin <35 g/L Score 1 CRP>10 mg/L and albumin ≥35 g/L Score 1 CRP>10 mg/L and albumin <35 g/L Score 2	(236–238)
Modified Glasgow prognostic score (mGPS)	CRP ≤ 10 mg/L and albumin ≥35 g/L Score 0 CRP ≤ 10 mg/L and albumin <35g/L Score 0 CRP>10 mg/L and albumin ≥35 g/L Score 1 CRP>10 mg/L and albumin <35 g/L Score 2	(238–241)
C-reactive protein-to-albumin (CAR)	$\frac{CRP}{Albumin}$	(242–244)
Neutrophil-to-lymphocyte ratio (NLR)	$\frac{Neutrophil\;count}{Lymphocyte\;count}$	(245–248)
Derived neutrophil-to-lymphocyte ratio (dNLR)	$\frac{Neutrophil\;count}{White\;cell\;count-neutrophil\;count}$	(249–252)
Monocyte-to-lymphocyte ratio (MLR)	$\frac{Monocyte\;count}{Lymphocyte\;count}$	(248, 253–255)
Platelet-to-lymphocyte ratio (PLR)	$\frac{Platelet\;count}{Lymphocyte\;count}$	(256–258)
Systemic immune-inflammation index (SII)	*Neutrophil* * *PLR*	(259–262)
Aggregate index of systemic inflammation (AISI)	*Neutrophil* * *platelet* * *MLR*	(263)

CRP, C-reactive protein.

## Paraneoplastic syndromes

Understanding the local inflammation associated with cancer allows for a better understanding of the interaction between tumor cells, immune cells, stromal cells, and the soluble factors released by this system. The release of proinflammatory factors from tumor or stromal cells—in the form of soluble factors or those contained in exosomes—into the bloodstream alters the production of APPs, metabolites, and cells, which impacts organs and systems, causing the appearance of symptoms unrelated to the tumor itself, called paraneoplastic syndromes (6). Approximately 8% of cancer patients present one or more paraneoplastic syndromes. Paraneoplastic syndromes are classified as neurologic, rheumatologic, dermatologic, or hematologic, depending on the type of tumor (264, 265). The manifestation of paraneoplastic syndromes has detrimental effects on patient’s quality of life and outcome. In addition, some paraneoplastic syndromes may exhibit tumor-promoting capabilities (7). In this section, we will discuss the development of myelopoiesis and cachexia.

### Stimulation of myelopoiesis in cancer-related inflammation

One of the main targets of pro-inflammatory mediators in the bone marrow is a process known as hematopoiesis, whereby hematopoietic stem and progenitor cells (HSPCs) differentiate into mature blood cells. Under physiological conditions, denominated as steady-state hematopoiesis, local and external signals promote the retention of HSPCs in the bone marrow. For this purpose, the bone marrow stroma cells, including endothelial, vascular, and osteolineage cells and macrophages, participate in a well-orchestrated network to control the proliferation and differentiation of HSPCs (266). However, during acute systemic infection or inflammation, factors released from pathogens and damaged cells, as well as proinflammatory cytokines (IL-6, IL-1β, and TNF-α), impact HSPCs and stromal cells, causing the rapid mobilization and differentiation of HSPCs into myeloid cells, a process known as emergency myelopoiesis (267, 268).

In cancer, it has been demonstrated that tumors take advantage of emergency myelopoiesis to rapidly expand pools of myeloid-derived cells showing immune suppressive and tumor-promoting activities (266). These cells are known as MDSCs and can be classified as granulocytic (CD11b+CD14-CD15+) or monocytic (CD11b+CD14+CD15-) depending on the myeloid progenitor from which they were derived (269).

As mentioned above, the plasma concentration of proinflammatory cytokines, such as IL-1β, TNF-α, and IL-6, increases in several types of cancer due to the sustained inflammatory environment caused by the tumor and its stroma. These cytokines, along with other bioactive soluble factors, such as GM-CSF and G-CSF, travel freely in circulation or are stored in exosomes reaching the bone marrow, causing the proliferation, mobilization, and skewed differentiation of HSPCs towards myeloid cells (270). Reports on G-CSF have indicated that as the G-CSF level in the bone marrow decreases, the expression of maintenance molecules for HPSCs, such as CXCL12, osteopontin, Kit-1, angiopoietin, and vascular cell adhesion molecule 1 (271) increases, thus promoting their proliferation. In the bone marrow, IL-6 binds to IL-6R—which is mainly expressed on hematopoietic multipotent progenitors but not in short- or long-term repopulating HPSCs—suppressing the differentiation of lymphoid cells and stimulating myeloid differentiation (272). Marigo et al. demonstrated in mice that GM-CSF, G-CSF, and IL-6 in the bone marrow caused the activation of the master regulator of emergency granulopoiesis, the C/EBPβ transcription factor, which is responsible for the differentiation of HSPCs into MDSCs and their immunosuppressive activity (273). Initial studies in humans by Wu et al. showed that increased frequencies of HSPCs and granulocytic progenitors in different cancer types were found in cancer patient’s blood compared to those of healthy donors (274). The increase in these cell populations was negatively correlated with several clinical parameters, including the time to progression and the stage of the disease, suggesting that activated bone marrow myelopoiesis is a phenomenon that promotes tumor progression.

However, these proinflammatory cytokines are not only responsible for the expansion and differentiation of HSPCs in the bone marrow. In a mouse model of breast and lung cancer, Sayed et al. found that hematopoiesis was biased towards myelopoiesis due to the action of TNF-α released from infiltrating CD4+ T cells (275). During this process, TNF-α caused a skewed differentiation of HSPCs towards MDSCs, with a concomitant decrease in erythroid and lymphoid precursors (275). In this scenario, TNF-α acted on HSPCs and MDSCs through TNFR-2, upregulating the expression of the caspase-8 inhibitor c-FLIP and promoting their survival (276). In these works, it has been demonstrated that tumor-promoting inflammation has profound systemic effects during the advanced stages of cancer, causing biased hematopoiesis toward the production of immunosuppressive myeloid cells and sustaining tumor progression. In addition, the results from these studies have helped to explain the altered percentages of hematological inflammatory parameters detected in cancer patients by the effect of these pro-inflammatory cytokines and growth factors in the bone marrow.

In recent years, owing to the use of NGS approaches and elegant *in vivo* models, it has been shown that hematopoiesis is altered and biased towards myelopoiesis in the early stages of tumor development (277). Surprisingly, pro-inflammatory cytokines play a significant role during this process by activating hematopoiesis in the bone marrow to direct the production of myeloid progenitors and micro-RNAs (miRNAs) delivered from the tumor. The TME plays a critical role in controlling this process. Some of these tumor-associated miRNAs, such as miR-23b-3p, miR-27a-3p, and miR-671-5p, in bone marrow downregulate the expression of genes involved in B-cell receptor signaling and antigen processing. With this work, it is tempting to speculate that other subsets of non-coding RNA, in addition to miRNAs, are released from the tumor or its stroma to activate myelopoiesis in the bone marrow. However, studies to demonstrate this proposal are required.

In addition to proinflammatory cytokines and non-coding regulatory RNA, myelopoiesis is controlled directly or indirectly by the metabolites released from the tumor as part of the metabolic reprogramming of cancer cells (2, 278). For example, the direct control of myelopoiesis by metabolites involves the participation of oxysterols and desmosterol in the differentiation and expansion of MDSCs. These cholesterol metabolites bind to retinoic acid-related orphan receptors expressed in the cell precursors of MDSCs (278). They have been detected in distinct pathologies, such as obesity, metabolic disorder, diabetes, and cancer (279). Recent evidence from Vladimirov et al. demonstrated that colorectal cancer patients had significantly increased values of cholesterol serum precursors compared to healthy donors, suggesting that cholesterol metabolism is altered in cancer (280). This evidence allows us to speculate that such patients might present altered myelopoiesis and thus increased numbers of MDSCs and other immunoregulatory cell populations; however, intensive research using *in vitro* and *in vivo* models in distinct types of cancer is needed to confirm this hypothesis. The activation of aerobic glycolysis indirectly affects cancer metabolic reprogramming in myelopoiesis. In a triple-negative breast cancer mouse model, Li et al. demonstrated that aerobic glycolysis stimulated the release of G-CSF and GM-CSF *via* C/EBP-β and liver-enriched activator protein. This glycolytic-dependent production of G-CSF and GM-CSF was correlated with increased MDSCs and low T lymphocyte counts in breast cancer patients (281). Although these results are pioneering, more studies are necessary to uncover the complete impact of altered metabolism on myelopoiesis in cancer.

Recent studies have demonstrated that hematopoiesis can occur outside the bone marrow under distinct inflammatory conditions, a phenomenon known as extramedullary hematopoiesis (269). In cancer, extramedullary hematopoiesis is mainly found in the liver and spleen, where HPSCs or myeloid progenitors arrive (282). In this setting, the accumulation of immunosuppressive cells, such as MDSCs, Tregs, and erythroid progenitor cells, in the spleen was found in a mouse model of breast cancer. Several reports have demonstrated that splenectomy in animals bearing tumors is associated with a decrease in the number of peripheral MDSCs cells and cytokines related to HPSCs mobilization (9, 283). In this scenario, a survival benefit was achieved due to decreased tumor growth following the splenectomy, suggesting that extramedullary hematopoiesis has a critical role in sustaining tumor growth and progression. However, in humans, a concomitant splenectomy following colon, liver, gastric, and pancreatic cancer has not shown any beneficial effects on patient’s overall survival (284). Nevertheless, owing to technical and experimental barriers, little is known about this process in humans; thus, most of the studies in this field are performed using animal models. For this reason, further research exploring the implications of extramedullary hematopoiesis in solid human tumors is required.

In solid tumors, the shift in anti-tumoral immunity towards a pro-tumoral response not only depends on the reshaping of the local tumor microenvironment by cytokines, chemokines, metabolic by-products, exosomes, or other released factors. The development of populations showing immune regulatory functions occurs in distinct and distant organs away from the primary lesion due to the systemic release of the factors mentioned above. In this setting, a considerable increase in the production of MDSCs in the bone marrow and secondary lymphoid organs and their subsequent migration to the tumor support the development of some cancer hallmarks by providing growth and angiogenic factors, cytokines, chemokines, and extracellular matrix remodeling enzymes (285) (**Figure 1**). In addition, these MDSCs strengthen the immunosuppressive tumor microenvironment by releasing immunomodulatory cytokines, metabolites, and other soluble factors and by expressing immune checkpoints on their surface, thus causing the recruitment of M2 macrophages, N2 neutrophils, and Tregs (286).

### Cachexia in cancer-related inflammation

Advanced tumors release a spectrum of factors that induce systemic inflammation, which is reflected in paraneoplastic syndromes, such as cachexia. Cancer cachexia is a multifactorial syndrome characterized by progressive sarcopenia that may be accompanied by a loss in adipose tissue. This pathology involves a harmful protein and energy balance and an abnormal metabolism due to anorexia. Cachexia cannot be reversed entirely with nutritional support, resulting in a detriment to the quality of life of cancer patients (287). In addition, cachexia is responsible for 20–30% of deaths in patients with advanced cancer. Tumors in which cachexia may develop include pancreatic, gastric, colon, lung, Hodgkin’s and non-Hodgkin’s lymphoma, breast, sarcoma, and leukemia (288).

The cellular mechanisms that induce cachexia are not yet fully understood; thus far, it is known to be a syndrome that involves several organs and is orchestrated at the systemic level by inflammatory factors released by the tumor and its microenvironment. Although systemic inflammation is not considered in the current classification as a parameter for diagnosing cancer cachexia, several authors have considered the measurement of inflammatory markers, such as CRP and IL-6, at the blood level (289). Biswas and Acharyya (290) mentioned that factors secreted directly by the tumor and non-tumor cells of the neoplastic microenvironment can mediate cachexia and other syndromes. These factors can directly interact with various tissues, such as muscle, liver, brain, and adipose tissue, and can induce metabolic reprogramming, leading to a negative metabolic energy state at the systemic level.

As mentioned above, as tumors continue to proliferate, the lack of oxygen and nutrients induces hypoxia. Hypoxia generates critical changes, for example, the metabolic turnover of tumor cells for energy. A hypoxic microenvironment releases several factors, including VEGF (291), which induces angiogenesis and recruits inflammatory cells, such as macrophages. In addition to the above components of the tumor microenvironment, the release of DAMPs by dead cells increases inflammation and generates extra-tumoral cytokines. Another important metabolite in cancer that contributes to tumor development and results from metabolic turnover is lactate (292, 293). The hypermetabolism of tumor cells leads them to consume large amounts of glucose and excrete lactic acid, which has pleiotropic activity, is involved in energy metabolism, has an immunosuppressive function, and promotes angiogenesis (294).

Lactate in the liver is converted to glucose in a process called gluconeogenesis. The glucose generated can be utilized by both the tumor cells and the host organism, increasing blood glucose. This hyperglycemia leads to insulin resistance, which is related to muscle wasting. The relationship between the tumor and liver *via* lactate is called the Cori cycle and is considered as a futile cycle in cancer cachexia (292). Insulin resistance in patients with cachexia, as well as in murine models, has been associated with muscle wasting and is induced by TNF-α (295). Noguchi et al. found a high correlation between TNF-α expression in muscle tissue obtained from the intestine of cancer patients and insulin resistance and muscle wasting. After tumor resection, the patients showed complete improvement in insulin resistance (296). More recent studies using a murine model of C-26 colon adenocarcinoma cell cachexia showed that insulin resistance occurred before weight loss. In the quadriceps muscle of these mice, the expression of four muscle atrophy-promoting genes, Atrogin-1, and MuRF-1, ubiquitin ligases E3 and Bnip3, was increased (297). Systemic inflammation is also associated with skeletal muscle wasting during cancer cachexia. The ubiquitin-proteasome pathway is activated during cachexia and is responsible for the degradation of most skeletal muscle proteins. Type IIB muscle fibers in an ApcMin/+ mouse model, which produced colon polyps, were shown to be highly susceptible to IL-6-mediated muscle wasting, as it induced the overexpression of the Atrogin-1 gene (298). In mouse bladder and colorectal cancer models, increased TNF-α, IL-6, and IL-1 in circulation and decreased muscle mass were associated with high Atrogin-1 and MurRF1 expression (299).

Among the factors that tumors can release are exosomes, which may contain molecules that promote muscle wasting. In a mouse model of cachexia, a group of researchers found that cachexigenic tumors released Hsp70/90 heat shock proteins into extracellular vesicles; furthermore, they participated in muscle wasting through TLR4 activation (300). Similarly, miR-181a-3p found in exosomes from a conditioned medium of oral squamous cell carcinoma induced endoplasmic reticulum stress and skeletal muscle wasting (301). Another study showed that HMGB1 protein, a DAMP contained in the exosomes of CT26 mouse colon cancer cells, induced the expression of the Atrogin1 and MuRF1 genes, leading to muscle wasting through the activation of TLR4/NF-κB axis (302). Another factor in exosomes that promotes muscle wasting is growth differentiation factor 15 (GDF-15), identified in the exosomes of CT26 colon cancer cells. GDF-15 can interact with C2C12 myotube cells, regulate Bcl-2/caspase, and induce cell apoptosis, favoring muscle atrophy in cancer cachexia (303).

The insulin resistance and systemic inflammatory cytokines in cancer cachexia patients also impact adipose tissue (304). In adults, adipose tissue is composed primarily of white adipocytes (WAT), although brown adipocyte fat deposits (BAT) exist in specific anatomic locations, such as the perivascular viscera and periviscus. BAT can also be found in supraclavicular, axillary, and inguinal subcutaneous fat and the intestinal walls (305). The difference between WAT and BAT is that the latter contain many mitochondria expressing uncoupling protein-1 (UCP-1). This molecule “uncouples” an electron in the process of ATP synthesis *via* OXPHOS across the inner membrane of the mitochondrion, which generates heat (306). Virtually all fat ingested in food is stored as WAT. In states of excessive exercise or prolonged fasting, triglycerides in WAT are degraded by three lipases: adipose triglyceride lipase (ATGL), hormone-sensitive lipase (HSL), and monoacylglycerol lipase (MGL). The regulation of lipases is highly influenced by several hormones, such as insulin, catecholamines, and growth hormone (307, 308).

In cancer cachexia, various cytokines, such as TNF-α and IL-6, promote the lipolysis of triglycerides in WAT (309). Shaw and Wolfe found increased lipolysis associated with increased blood fatty acids in patients with cachexia (310), indicating that lipid metabolism is dysregulated in cachexia states. In a muscle stem cell model, as well as in models of cachexia induced by human kidney neoplasia in mice, researchers observed that the process of muscle atrophy was preceded by an increase in FA β-oxidation as well as inflammatory factors, such as IL-1 β, IL-6, IL-8, and TNF-α. In this work, blocking FA β -oxidation using a carnitine-palmitoyltransferase-1 inhibitor also blocked muscle wasting (311). Another mechanism of adipose tissue degradation is the browning of WAT. For a long time, it was believed that the function of WAT was lipid storage, while BAT was involved in heat dissipation and the regulation of temperature (312). It is now known that WAT can be converted to BAT *via* specific mechanisms, such as hypothermia. The browning of WAT has been associated with cachexia.

Petruzzelli et al., using several genetically engineered mouse models, observed that the browning of WAT was an event that preceded muscle wasting. They also showed that IL-6 and catecholamines increased UCP-1 expression in the WAT of these cachexic mice (312). Recently, a study in cancer patients with and without cachexia showed an association between tumor-derived factors and inflammatory changes in the adipose tissue of the cachectic patients (313), suggesting that factors released by the tumor and its microenvironment modify adipose tissue metabolism. Exosomes have been found to play an important role in adipose tissue wasting.

Exosomes derived from cell lines and the plasma of gastric cancer patients contain ciRS-133, which induces the browning of WAT (314). Exosomes derived from lung adenocarcinoma cell lines containing TGF- β were shown to inhibit adipogenesis in primary adipocyte cultures from healthy subjects (315). The miR155 in gastric cancer exosomes was shown to promote the browning of adipose tissue through the transcription factor CCAAT/enhancer-binding protein β, which upregulates UCP1 (316).

Finally, circulating inflammatory cytokines produced by the tumor microenvironment affect the central nervous system, amplifying and orchestrating the symptoms of cancer-associated cachexia and causing anorexia, fatigue, and the wasting of muscle and fat tissue. In particular, a loss of appetite has been associated with hypothalamus inflammation. The nucleus of the hypothalamus regulates energy homeostasis (290, 317, 318). Increased cytokine expression in the brain alters the neurochemistry of the hypothalamus nucleus, where cytokines activate pro-opiomelanocortin (POMC) and cocaine- and amphetamine-regulated transcript (CART) neurons, which mediate satiety and reduce food intake. The activation of these neurons induces serotonin release, suppressing appetite. In addition, cytokines are likely to inhibit neuropeptide Y (NPY) and agouti-related peptide (AgRP) neurons, which mediate appetite and energy intake. These changes in the neurochemistry of the hypothalamus result in a “resistance” to signals that inform the brain of energy deficits in the periphery. As mentioned above, adipose tissue wasting leads to the circulation of free FA, which generates a satiety signal in the hypothalamus, contributing to anorexia (290, 317, 318). On the other hand, some reports have indicated that the stimulation of the hypothalamic–pituitary–adrenal axis with IL-1 induces the release of glucocorticoids that act on skeletal muscle and accelerate protein degradation (317, 319, 320).

Cancer patients present two types of damage: that produced locally by the tumor, which can be direct damage to the organ where it is located, and immunopathological damage that occurs when the tumor and the tumor microenvironment release compounds that cause metabolic derangement and systemic inflammation, such as in cachexia. IL-1, IL-6, and TNF-α play an essential role at the systemic level as inducers of cancer cachexia (321). In the 1970s, although there was not yet a methodology with sufficient sensitivity to measure these cytokines in cancer patients and obtain consistent results, treatment with anti-TNF- and IL-6 antibodies was proven to be effective in reducing cachexia in mouse tumor models (322).

Jafri et al. proposed a cachexia index to estimate the degree of cachexia in patients with advanced non-small-cell lung carcinoma (NSCLC) and to identify which patients might respond to cancer cachexia treatment (323). This index considers albumin; the skeletal muscle index, which results from comparing abdominal and paraspinal muscle scans between the time of diagnosis and one month later; and the NLR (323). In another paper, a sarcopenia index was defined as the muscle area at the third vertebra/height^2^, and values of ≤ 55 cm^2^/m^2^ for men and ≤ 39 cm^2^/m^2^ for women indicated sarcopenia. This index was correlated with CRP and the neutrophil/lymphocyte ratio in patients with small-cell lung carcinoma. Sarcopenia was found to have a linear relationship with CRP (324). Finally, Barrer et al. found that the neutrophil/lymphocyte ratio was associated with weight loss in patients with colon, lung, and prostate cancer cachexia (325).

The cachexia syndrome is characterized by the metabolic dysregulation of carbohydrates, lipids, and proteins in various organs and the sympathetic activation of the nervous system. All this leads to a poor quality of life for patients, and their diminished physical condition may not be suitable for treatment. The incidence of the anorexia–cachexia syndrome is high in cancer patients, affecting the evolution of the underlying disease at the clinical level. Unfortunately, its clinical management is complex in cancer patients. Consequently, cancer patients severely compromised nutritional status and weight loss remain standard features. It is essential to recognize and treat this syndrome early, together with antitumor therapy, to prolong survival and positively influence the quality of life of cancer patients.

## Concluding remarks and perspectives

During the uncontrolled growth of tumors, several inflammatory factors, including—but not limited to—cytokines, chemokines, growth factors, metabolites, and ncRNAs, are produced and released by both tumor and stromal cells. The continuous release of these factors causes impacts at the local level; their delivery into the bloodstream reaches other systems or organs, such as the liver, nervous system, bone marrow, adipose tissue, skeletal muscle, etc. In this setting, the continuous presence of these factors, in particular IL-6, IL-1, TNF-α, G-CSF, and GM-CSF, promotes cancer-associated systemic inflammation. Due to their pleiotropic activity, these molecules impact distinct subsets of cells, such as endothelial, epithelial, mesenchymal, neurologic, and hematologic cells, amplifying the inflammatory state and the clinical manifestations of the aberrant function of organs and systems, known as paraneoplastic syndromes. Paraneoplastic syndromes have detrimental effects on the patient’s quality of life and can sometimes cause their demise.

In addition, paraneoplastic syndromes can be exacerbated during the administration of cytotoxic antitumor therapies focused on eliminating tumor cells. Because advanced-stage tumors deregulate communication between the immune, endocrine, and neurological systems, a deeper we believe that, in addition to using antitumor agents, knowledge of the regulation of neuro–endocrine–immune intercommunication during cancer-associated inflammation will to could favor the development of upcoming therapies that will impact patient survival and quality of life. In support of this proposal, recent reports have demonstrated that nonsteroidal anti-inflammatory drugs may be used in cancer to reduce systemic inflammation (326). However, in clinically advanced tumors, cancer-associated systemic inflammation is dysregulated at another level; thus, these anti-inflammatory drugs would have no effect. Furthermore, the use of steroid drugs, such as glucocorticoids, could suppress the antitumor immune response, as they block the function of CD8+ effector T and NK cells (327). To address this issue, models that capture the complexity of tumor–host organ interactions will help clarify the picture and offer new therapeutic alternatives to improve patient outcomes.

## Author contributions

Conceptualization and design of the entire manuscript and draft: DA-C, RC-D, MM-M, and JL-G. JL-G, DA-C, and MM-M wrote the microenvironment section. JL-G, RC-D, and AC wrote the participation of the immune response in cancer section. DA-C, RC-D, JB-L, and MP-M wrote the systemic tumor microenvironment-derived cytokines section. JL-G, UR-N, and MM-M wrote the parameters to assess systemic immune-inflammatory markers section. DA-C, RC-D, and AC wrote the paraneoplastic syndromes section. **Figure 1** was designed by RC-D, DA-C, and JB-L. **Tables 1**, **2** was designed by JL-G, RC-D and MM-M. All authors contributed to the article and approved the submitted version.

## Funding

This manuscript was partially funded by Consejo Nacional de Ciencia y Tecnologia (CONACYT) (grant number: 284775).

## Acknowledgments

The authors acknowledge Instituto Nacional de Enfermedades Respiratorias Ismael Cosio Villegas. RC-D, JB-L, MM-M, and MP-M are students from the Posgrado en Ciencias Biologicas Universidad Nacional Autonoma de Mexico, Mexico and Posgrado en Ciencias en Biomedicina y Biotecnologia Molecular, Instituto Politecnico Nacional, Mexico, Mexico, respectively. All are recipients of a fellowship from CONACYT (RC-D 631047) (JJB-L 1085486) (MM-M 718959) (MP-M 740805).

## Conflict of interest

The authors declare that the research was conducted in the absence of any commercial or financial relationships that could be construed as a potential conflict of interest.

## Publisher’s note

All claims expressed in this article are solely those of the authors and do not necessarily represent those of their affiliated organizations, or those of the publisher, the editors and the reviewers. Any product that may be evaluated in this article, or claim that may be made by its manufacturer, is not guaranteed or endorsed by the publisher.
